# Loricrin at the Boundary between Inside and Outside

**DOI:** 10.3390/biom12050673

**Published:** 2022-05-06

**Authors:** Yosuke Ishitsuka, Dennis R. Roop

**Affiliations:** 1 Department of Dermatology Integrated Medicine, Osaka University Graduate School of Medicine, 2-2 Yamadaoka, Suita 565-0871, Osaka, Japan; 2 Department of Dermatology and Charles C. Gates Center for Regenerative Medicine, University of Colorado Anschutz Medical Campus, Aurora, CO 80045, USA; dennis.roop@cuanschutz.edu

**Keywords:** loricrin, ichthyosis, reduction, oxidation, Langerhans cell, TGF-β

## Abstract

Cornification is a specialized mode of the cell-death program exclusively allowed for terrestrial amniotes. Recent investigations suggest that loricrin (LOR) is an important cornification effector. As the connotation of its name (“lorica” meaning an armor in Latin) suggests, the keratin-associated protein LOR promotes the maturation of the epidermal structure through organizing covalent cross-linkages, endowing the epidermis with the protection against oxidative injuries. By reviewing cornification mechanisms, we seek to classify ichthyosiform dermatoses based on their function, rather than clinical manifestations. We also reviewed recent mechanistic insights into the Kelch-like erythroid cell-derived protein with the cap “n” collar homology-associated protein 1/nuclear factor erythroid 2-related factor 2 (NRF2) signaling pathway in skin health and diseases, as LOR and NRF2 coordinate the epidermis-intrinsic xenobiotic metabolism. Finally, we refine the theoretical framework of cross-talking between keratinocytes and epidermal resident leukocytes, dissecting an LOR immunomodulatory function.

## 1. Introduction and Overview

### 1.1. Cornification: A Successful Tissue Adaptation to Land

The body’s surface is covered with a specialized protective layer called the epidermis, from the scalp to the foot: the stratified squamous epithelium consisting of keratinocytes (KCs). Mammalian epidermises acquire specialized apparatuses to accommodate various external stresses, including physical injuries, harmful substances, or ionizing radiation. The stratum corneum (SC) overlies most parts of the epidermis (interfollicular epidermis (IFE)) and protects against dehydration or mechanical loads. Interspersed in the epidermis are skin appendages that also produce cornified materials. The hair provides the integument with insulation against heat/cold or trauma in mammals, and comprises the hairy (fibrillar) attachment system in the reptilian foot [1]. The mammalian nail comprises essential components of the haptile, tactile, mechanic, and the defensive and/or predatory instrumentarium [2].

These end-products are derived from the functional cell-death program termed cornification [3]. The IFE and appendages serve different functionalities by producing the SC (armor) and the hair/nails (instruments), respectively. The effector proteins (keratins and keratin-associated proteins) exhibit various characteristics to meet the biophysical/biochemical properties required for specific body parts. Despite the substantial difference in appearance, they are the macromolecular “protein alloy”, comprising the extensive intra/intermolecular formation of ε-(γ-glutamyl) lysine isopeptide [4,5]/disulfide (–S–S–) cross-linkages [6,7]. Noteworthily, these vital biochemical characteristics are observed even in the simple columnar epithelium, covering most parts of the wet-surfaced digestive tract. It was recently proven that gel-forming mucins (Mucin 2, MUC2), secreted from the goblet cells, undergo transglutaminase 3 (TGM3)-mediated isopeptide cross-linkages [8], which are resistant to reducing reagents that cleave disulfide (–S–S–) cross-linkages [9]. The results are a reminder of the very early observations that the cell membrane-like remnants of the “mummified” KC (corneocyte), i.e., cornified cell envelopes (CEs), are highly resistant to exhaustive hydrolysis [10] because of their extremely strong isopeptide cross-linkages [5]. Therefore, surface protection by turning secreted substances into macromolecules may be a strategy conserved across tissues.

Analogously to the ontogenetic evidence, vertebrates have acquired gene sets that encode the effector proteins more suitable for cornification than the pre-existing repertoire through gene duplication or deletion during evolution [11,12]. For instance, when amphibians invaded arboreal habitats on the waterside [13], ancestral keratins are thought to have emerged as constituents of the toe pad, which endure the forces caused by the repetitive contact with the waterside environment [13,14,15]. Thus, the ancient keratins are considered to have evolved into a major group of cysteine-rich alpha keratins, enabling the hairy (fibrillar) attachment system in reptiles [1] and constituting mammalian hair [13]. Despite the presence of amphibian CEs [14] or cornified layers [16] in the epidermis, phylogenetic evidence strongly supports the notion that cornification is an ultimate form of body wall protection that is exclusively allowed for terrestrial amniotes (reptiles, birds, and mammals) [17,18,19,20,21]. A major cornified cell envelope protein, loricrin (LOR), is considered to have originated in a common ancestor of modern amniotes, along with the sauropsid-specific corneous beta-proteins (beta keratins) that constitute the reptilian/avian SC [12,21]. Aggregately, the major product of cornification SC is arguably an example of successful tissue adaptation to land.

### 1.2. Cornification in Skin Health and Diseases

Several diseased conditions of inflammatory or non-inflammatory causes comprise aberrations in cornification. Psoriasis, eczema (atopic dermatitis (AD)), or acne may exemplify the former, and calluses, corns, or ichthyoses may represent the latter. As a higher-order structure with dynamics, the epidermal tissue produces pro-inflammatory cytokines in response to primary injuries, and direct pathogen recognition via the toll-interleukin receptor (TIR) [22]. This frontline protective immunity can provide critical directions for such diseases. Indeed, therapeutic intervention in the tissue response cascades has been successful for AD and psoriasis by targeting the interleukin (IL)-4/IL-13 receptor and the IL-23/IL-17 signaling pathway, respectively [23]. Omic approaches, such as single-cell technology, have provided us with high-resolution images of cutaneous pathologies [24]. Data-mining approaches may boost the identification of potential therapeutic targets and depict the evolutionary trajectory of various diseased tissues, including skin cancers [25] or inflammatory conditions [26]. Dissecting the cause–effect relationships between the diseases and the genetic predisposition factors, including the loss of structural proteins (e.g., filaggrin (FLG)-deficiency (ichthyosis vulgaris—IV) in AD [27]) or unchecked TIR signaling cascades (e.g., hyperactivated caspase recruitment domain family member 14 (*CARD14*) in psoriasis [28]) may remind us of the premise that most ailments are products of gene–environment mismatches [29]. In this article, we focused on LOR and NFE2-related factor 2 (NRF2), which are important cornification effectors in IFE, and sought to revise the theoretical framework of the cross-talk between IFE KCs and leukocytes proposed previously [30,31,32,33].

## 2. SC Permeability Barrier: The Priority

### 2.1. IFE Cornification in Brief

In the homeostatic status, lineage-committed IFE KCs [25] exit from the proliferative layer (stratum basale—SB), detach from the basement membrane [34], and migrate upward through the stratum spinosum and the stratum granulosum (SG). SG is morphologically identified by the presence of keratohyalin granules, and contains keratin-associated proteins FLG and LOR [35]. Tight junctions (TJs) connect the plasma membrane of adjacent KCs and form a belt-like structure (zonula) [36], denoting the apical end of the living epidermal layers. The SG comprises three distinct layers arranged in an apicobasal direction. The uppermost SG1 is particularly important because IFE KCs undergo the specialized cell-death program cornification (or “corneoptosis” [37]). The specialized body wall defense program involves a myriad of biological processes. These include the transient influx of calcium (Ca^2+^) [38], intracellular acidification [37], autophagy/nucleophagy [39], activated proteases [40,41,42,43,44], vitamin A (VA) metabolism [45]/vitamin D metabolism [46], the lamellar granule (LG)-mediated secretion of intracellular content (lipids/proteins [47,48]) to the interstitial space [49], the fusion of LG-limiting membranes with apical plasma membranes [50,51], TJ disappearance [36], dephosphorylation of the huge pro-FLG protein and the subsequent protease-mediated breakdown into FLG monomers (reviewed in ref [52]), TGM-mediated cytoskeletal cross-linkages [53,54] and disulfide (–S–S–) cross-linkages [55,56,57,58,59,60,61,62], etc.

After leaving the transitional layer, IFE KCs turn into a polymerized epithelial sheet composed of “mummified” KCs (corneocytes). Corneocytes are surrounded by orderly arranged lipids (ceramides) covalently attached to the outside of CEs (corneocyte lipid envelopes (CLE)), and the structure of the SC is analogized to “bricks and mortar” [63]. Despite being “dead”, the lower layer (stratum compactum) appears important, since corneocytes continue structural maturation before shedding off the skin surface. This process involves cross-linkages of cytoskeletal and junctional components (desmosomal-keratin scaffold (DKS) of the SC) with CEs. The former includes differentiated keratins K1/K10 and FLG monomers, and the latter corresponds to corneodesosin (CDSN). The major CE protein, LOR, moves to the desmosomal area at the periphery [64,65], reinforces the DKS [66] via the formation of intermolecular disulfide (–S–S–) cross-linkages, promotes corneocyte compactization/flattening, and transitions into the stratum disjunctum, ready for peeling away [56,57,67].

### 2.2. FLG: A Precursor of Natural Moisturizing Factors

The relatively immature stratum compactum appears to absorb small water-soluble molecules with the help of humectants (so-called natural moisturizing factors (NMFs) [52]) stored in corneocytes. Because the FLG monomers are major precursors of hygroscopic (moisture-absorbing) amino acids, such as arginine (Arg) [52], they possess a much smaller amount of Arg in the stratum compactum and fail to confer impermeability against small water-soluble molecules that could be absorbed by corneocytes, although FLG-knockout mice exhibited an abnormal aggregation of keratins, as the name connotes (i.e., filament aggregation [67]). Metal ions are the established water-soluble substances that cause allergic contact dermatitis (ACD). Consequently, FLG-deficient mouse models exhibited hypersensitive reactions [68,69] similar to IV patients [70], and were susceptible to *Staphylococcus aureus* (*S. aureus*) colonization [71]. The stratum compactum may correspond to the aseptic mucous layers composed of MUC2 polymers that prevent the penetration of commensal bacteria and subsequent T helper (T_H_) 17-mediated immunopathology that models inflammatory bowel diseases [8].

### 2.3. Corneodesmosomes: The Regulator of Corneocyte Cohesion

The SC is a polymerized sheet consisting mainly of keratins and keratin-associated proteins. In addition to cross-linkage and the subsequent breakdown of FLG monomers, desmosomal components must be degraded quickly [72,73]. Serine proteases, SC chymotryptic enzymes (kallikrein 7—KLK7), and SC tryptic enzymes (SCTE; KLK5) target desmosomal components [74]. A serine protease inhibitor, lymphoepithelial Kazal-type related inhibitor type 5 (LEKTI), antagonizes KLK activity and prevents the premature proteolysis of the N-terminal extracellular domain of desmoglein 1 (DSG1)/CDSN. Unopposed desmosomal breakdown leads to postnatal lethality in mice [40,43,75,76,77,78] and severe allergic manifestations that include AD-like skin phenotypes in humans [79,80]. Likewise, the loss-of-function (LOF) variants in DSG1/CDSN phenocopy the allergic symptoms in humans (reviewed in [81] and discussed later). Although a defective TJ-based permeability barrier can cause ichthyosiform skin phenotypes in humans [82], this aggregated evidence suggests that the DKS of the SC [66] assures successful cornification.

### 2.4. A classification of Ichthyosiform Dermatoses Based on Gene Functions

The aggregated molecular genetic evidence regarding rare, autosomal recessive ichthyosiform dermatoses suggests the importance of “the mortar” for preventing dehydration. We took autosomal recessive congenital ichthyosis (ARCI), peeling skin syndrome-1 (PSS1) (Online Mendelian Inheritance in Man (OMIM) #270300, accessed on 1 April 2022 [83]), Netherton syndrome (NS; OMIM #256500) [79], skin dermatitis, multiple severe allergies, and metabolic wasting (SAM) syndrome (OMIM #615508) [80] as clinical examples. Although Vohwinkel syndrome (VS) with ichthyosis (OMIM #604117) [84] is not an autosomal recessive trait, we took the rare autosomal dominant dermatosis to clarify the LOR effector function in cornification.

#### 2.4.1. ARCI

ARCIs are heterogeneous groups of rare and congenital ichthyoses typically characterized by a parchment-like collodion membrane at birth and subsequent widespread hyperkeratosis covering almost the entire body surface (reviewed in [85]). Hyperkeratosis severity may vary depending on the afflicted individual, ranging from skin flakiness (congenital ichthyosiform erythroderma, CIE) to thick, large, and plate-like squames (lamellar/harlequin ichthyoses, LI/HI). Although there are no established classifications based on gene functions, defects in the following biological processes have been suggested: i. lipid metabolism (ceramide synthesis) [86,87,88,89,90,91,92,93]; ii. LG function [94]; iii. CLE formation [95]; and iv. FLG processing [96,97] (Table 1).

#### 2.4.2. Desmosomal Defects Accompanying Allergic Manifestations

NS [79] and PSS1 [98] are similar in that CDSN deficiencies cause both conditions, by unopposed degradation and absence, respectively. LOF variants in the *DSG1* gene cause SAM syndrome [80] (Table 2).

The molecular pathology of these skin-peeling disorders provides a profound insight into AD pathogenesis [81]. NS is clinically characterized by ichthyosiform erythroderma, hair shaft abnormality, and atopic manifestations. The patients are born with erythroderma that develops into ichthyosis linearis circumflexa (ILC) [99]. Systemic complications are also common, including failure to thrive (FTT), eosinophilia, increased serum IgE levels, systemic infection, and food allergies [81]. LOF variants cause NS in the serine protease inhibitor of the Kasal-type 5 (*Spink5*) gene that encodes LEKTI. Unchecked proteolytic activity of KLKs promotes CDSN/DSG1 extracellular domain degradation [81]. Recapitulating the lethal phenotype results in SC detachment [43], and forced KLK5 overexpression in the epidermis faithfully replicated major NS symptoms in mice [40]. LOF variants cause PSS in the *CDSN*, clinically characterized by ichthyosiform erythroderma, FTT, increased IgE levels, eosinophilia, asthma, urticaria/angioedema, and food allergies [81]. Currently, two independent gene knockout studies of CDSN have been reported [77,78], both resulting in postnatal lethality that phenocopies the *Spink5*-knockout mice. LOF variants cause SAM syndrome in the *DSG1* gene, clinically characterized by striate palmoplantar keratoderma (PKK), FTT, and atopic manifestations such as NS or PSS1. SAM syndrome-associated PKK is a distinctive clinical feature among these desmosomal disorders, suggesting that DSG1 serves as an indispensable component of the DKS in the differentiating epidermal layer, which, along with the K1/K10 pair [100], resists shear stress in the weight-bearing skin, i.e., the palm and sole [101]. DSG1-knockout mice exhibit postnatal lethality due to a superficial detachment of the epidermal tissue [75,76], phenocopying the NS/PSS1 mouse models [40,43,78,79].

#### 2.4.3. Ichthyosiform Dermatosis Caused by LOR Mislocalization

VS with ichthyosis is an autosomal dominant ichthyosiform dermatosis caused by *LOR* gene variants [84]. Characteristics include palmoplantar hyperkeratosis with small “honeycomb” depressions and the progressive formation of pseudoainhum (digital constricting bands) [84] or erythrokeratodermia [102]. VS-related *LOR* mutations result in delayed translation termination, and the C-terminal glycine- or glutamine/lysine-rich domain is replaced with arginine and leucine-rich amino acid sequences. In contrast to wild-type (WT) LOR that moves to the cell periphery and forms CE [35], mutant LOR accumulates in the nucleus [102]. However, LOR glutamine/lysine residues required for TGM-mediated isopeptide cross-linkages should not be affected by the VS mutation located at the C-terminus [60]. Indeed, the LOR C-terminal mutation generates a nuclear localization signal, and abnormal nuclear accumulation of the LOR perturbs normal cornification, causing an impaired SC permeability barrier assessed by transepidermal water loss or the passive diffusion of water-soluble Lucifer yellow fluorescent dye [103]. We have previously demonstrated the dominant negative effect of the vs. mutation by generating the VS-Lor-transgenic mice with an LOR-knockout (LKO) background, which phenocopied VS-Lor-transgenic mice with WT LOR [103] (Table 3).

#### 2.4.4. Potential Targeted Therapeutics Based on the Gene Expression Profiles

Despite clear cause–effect relationships proven by molecular genetic studies, treating ichthyosiform dermatoses remains unsatisfactory [104]. Most conditions are treated with supportive regimens (skin moisturizers or emollients) or VA derivatives [104]. As in the proposal of “psoriasiform ichthyosis”, referring to NS/ILC [105], extensive gene expression profiling using relatively small skin biopsy or blood samples has uncovered T_H_17-skewing as an immunological signature shared across ichthyosiform dermatoses [104,106].

The IL-23/IL-17 axis executes body surface pathogen clearance through the prompt recruitment of neutrophils [107], with psoriasis (psoriasiform tissue reactions [108]) being an epitome of such tissue responses. Therefore, one might have expected the results from the omics approach, given that defects in the epidermal structure cause most ichthyosiform dermatoses and can activate the primary immune defense of the skin (so-called “preemptive” immunity [109]) imprinted within the tissue [110,111,112]. Although DSG1-knockout mice exhibit postnatal lethality due to a superficial detachment of the epidermal tissue [75,76], they exhibit T_H_17-associated inflammatory signatures on embryonic day 18.5 (right before birth) [75]. Thus, results from the rodent models and SAM syndrome patients may correspond to immunological alertness [109] augmented by the breach of superficial epidermal tissue [71]. In fact, therapeutic interventions on the IL-23/IL-17 axis in NS [113,114,115,116] or SAM syndrome [75,115] patients have shown success in clinical anecdotes. Based on a recent small-size pilot study, Guttman-Yassky and Paller et al. concluded that younger and more erythrodermic patients will benefit from these immunotherapies [115]. Future studies are needed to clarify endotypic or phenotypic differences in responders vs. non-responders. Clinical studies will increase our understanding of disease heterogeneity and lead to the precise prediction of therapeutic responses [115].

## 3. LOR as a Major Epidermal Differentiation Component

### LOR and NRF2 in IFE Cornification

Because LOR is a major CE protein appearing from the cytosol at the later phase of cornification and replacing the plasma membrane [50], the largely asymptomatic LKO phenotype might not have been surprising retrospectively [59]. We previously found that LKO mice exhibit a delay in the maturation of the epidermal permeability barrier assessed by Toluidine blue dye exclusion in utero [59]. The LKO embryo showed a massive upregulation of small proline-rich proteins 2 (SPRR2s) [58,59]. The *Sprr*/*SPRR* genes encoding the cytoprotective proteins reside within the epidermal differentiation complex (EDC) [117] located in the mouse chromosome 3/human chromosome 1, clustering with other cornification proteins, including *LOR*, involucrin, and *FLG* [30]. SPRR proteins are rich in thiol (–SH), show structural resemblance to LOR [118], and are recently shown to have antimicrobial properties in extracutaneous tissues, including the gut [119] and the kidney [120]. Owing to the abundance of thiol (–SH) residues, SPRR2s possess superior antioxidative capacity compared to LOR in vitro [121], which may help vertebrates recover from physical insults, including wounding [122], ultraviolet (UV) [123], or the invasion of metazoan parasites [119]. Along the same line, late-cornified cell envelope protein 1s (LCE1s) are also abundant in LKO CE [118]. Recently, it was proven in humans that LCE3 possess a β-defensin-like broad antimicrobial activity in vitro and, like SPRRs, they are massively induced following superficial epidermal injury [124] or psoriatic epidermis [125,126].

The Kelch-like erythroid cell-derived protein with the cap “n” collar homology-associated protein 1 (KEAP1)/NFE2-related factor 2 (NRF2) signaling pathway is a ubiquitous antioxidative machinery that drives quick antioxidative responses following the detection of cellular oxidative insults [127]. Actin-associated cytoskeletal KEAP1 protein [128] is rich in thiols (–SH), and subjects the transcription factor NRF2 to proteasome-mediated degradation in the cytosol in the steady state [127]. Electrophilic stresses or other non-specific injuries cause conformational changes in KEAP1 proteins, stabilizing the NRF2 protein and unleashing transcriptional activity [127]. Thus, KEAP1 inhibition leads to the constitutive activation of the transcription factor, NRF2, and causes extremely strong antioxidative responses in multiple organ systems [127]. However, the *Keap1*-null mutation in mice leads to postnatal lethality; an autopsy of pups revealed that the upper digestive tract (the esophagus–forestomach) was obstructed with keratinous materials, and the skin manifestation somewhat resembled that of ARCI [129]. Because KEAP1/NRF2 expression levels become higher upon epidermal differentiation [130,131], the system may have evolved to convert the cellular dysregulation of various causes, including electrophiles [132,133], physical injuries [129,134], or UV [131,135], into antioxidative responses. This evidence indicates that NRF2 is a strong keratinization regulator [129,136] and reminds us of the principle that sulfur metabolism governs epidermal differentiation [6,7].

The delayed barrier formation of LKO embryo and the supposed LOR function, i.e., a major TGM substrate that promotes disulfide (–S–S–) cross-linkages in the SC [61,62] and the compensatory upregulation of the antioxidative SPRR2s, prompted us to test the role of the KEAP1/NRF2 signaling pathway in the LKO epidermis [55,58,118,137]. Because LOR-NRF2 double-knockout (DKO) mice look apparently healthy [55], we introduced the dominant-negative *Nrf2* (Δ*Nrf2*) gene under the control of the *Lor* promoter to obtain differentiating layer-specific gene expression [58]. Compared with normal-looking WT-Δ*Nrf2* mice, LKO-Δ*Nrf2* mice exhibited postnatal lethal dehydration [58]. Correspondingly, we recently found that the DKO mice exhibit a significant delay in the recovery of the SC permeability barrier following SC removal [55]. As was previously shown in other experimental systems in which constitutively active NRF2 was overexpressed in the epidermis [138], NRF2 causes the upregulation of the corneocyte cohesion protein CDSN [55]. Perhaps more importantly, meticulous ultrastructural observations revealed that corneodesmosomes remained undegraded in LKO/DKO mice compared to WT/*Nrf2*-knockout mice, while CEs were thinner in LKO mice than in WT mice [55]. These results may correspond to the supposed LOR kinetics in vivo: i. moving to the cell periphery and desmosomal area [64,65]; ii. forming CE and promoting corneocyte maturation [55,56,57,59,61,62]; iii. connecting LG-derived CDSN to CE via disulfide (–S–S–) cross-linkages [67,139,140]. In short, LOR appears to be the fundamental component that promotes the terminal differentiation of the epidermis [33].

## 4. “Structural Imprinting” of the Cutaneous Immune Effector Functions

### 4.1. Breach of SC Permeability Drives Inflammation

We have stressed the importance of the SC permeability barrier for the defects in cornification. ARCI is caused by defects in the following: (i) lipid metabolism (ceramide synthesis); (ii) LG function; (iii) CLE formation; and (iv) FLG processing, while desmosomal defects cause allergic manifestations [81]. Regarding LOR function, we have characterized that LOR is dispensable for the SC permeability barrier per se, unless it is mislocalized in the nucleus [103]. Nonetheless, LKO CEs are substantially thinner than WT [55], abundant with the “scaffolding” components that form CLEs [57] and have incomplete K1/K10 cross-linkages ([57] and unpublished observations). Although LKO CE phenotypes faithfully mirror Steinert’s model of CE assembly [50], investigations regarding the mechanism that regulates the desmosomal disappearance or skin peeling (desquamation) were in a nascent stage [141]. Moreover, CE isolation from the human skin hampered the analysis of the epidermal differentiation dynamics, involving the cellular turnover. Therefore, retrospectively, the elegant model suffered from a paucity of information regarding how desmosomal components are broken down [50,142] (although desmosomal components are the initial “scaffolding” component [143]). Given that TGM1-knockout mice do not generate CEs due to impaired LOR polymerization at the cell periphery [54,139], it would have been reasonable that the largely asymptomatic phenotype observed in LKO mice was surprising [140]. A more recent characterization of lethal *Cdsn* [77,78]/*Dsg-1* [75,76]-knockout phenotypes suggests that corneodesmosomes are indispensable for successful cornification. An intriguing question arises here: why do defects in corneodesmosomal components cause allergic inflammation that resembles AD [81]? A recent *Dsg1*-knockout study suggests that this immunological skewing emerges as early as on embryonic day 18.5, when the epidermis fully matures, starts to turn over (degradation of the SC components: FLG or CDSN/DSG1), and becomes ready for the terrestrial life [144,145].

### 4.2. Epidermis-Directed Immune Responses following Breached Barrier Function

It is known that the epidermis is a source of danger signals, causing immunological alertness and subsequent tissue responses [146]. For instance, the microbiological milieu of epidermal inclusion cysts does not differ whether inflamed or not [147], presumably because of the SC’s properties evoking strong inflammatory responses. The SC causes neutrophilic abscess when subcutaneously implanted [148], and its aqueous extract causes pyrexia when administered intravenously [149] in mice. This strong SC pro-inflammatory property appears attributable to IL-1α, which is shown to be abundant in individuals with ichthyosis vulgaris (caused by LOF *FLG* variants) [150], or LI [151], in close association with the maturation/recovery of the epidermal barrier [152,153]. Although IL-1α and IL-1β are prototypes of leaderless cytokines lacking signal peptides for secretion, IL-1α does not require an inflammasome for activation [154]. The pro-IL-1α stored in the nucleus is also biologically active [155] and is released in response to physical injuries such as mechanical deformation [156]. Therefore, IL-1α, abundantly present in the SC, serves as a damage-associated molecular pattern, initiating ACD [157] or AD-like inflammation in the “flaky tail” mice [158]. It appears most likely that IL-1α, densely present atop the epidermis, informs epidermal damage (or irritation), conditions antigen presenting cells, and enhances inflammatory responses skewed from the acute T_H_17 type [159] toward the chronic T_H_2 type [132]. Indeed, Bermekimab, a monoclonal anti-IL-1α antibody, has potential as a new therapeutic option [160], inducing rash clearance and reducing itching in patients with AD (NCT03496974). The evidence suggests that, in response to injuries, the SC generates intrinsic inflammatory cues that break tolerance and promote T_H_2 immunological memory, a hallmark of AD [132].

### 4.3. LOR: The Scaffold of Sulfur Metabolism in the IFE

Next, we must return to the principle that cornification is all about anabolizing sulfur; thiol (–SH) is effectively converted into disulfide (–S–S–) [6,7], and TGMs or their substrates constitute important effectors [54,59,143]. LOR, precipitating at the cell periphery and replacing the plasma cell membrane [35,55], confers photoprotection in corneocytes [56] and protects against polyaromatic hydrocarbon 7,12-dimethylbenz (a) anthracene (DMBA) [137]. LOR absence/presence does not interfere with CLE formation [63,161], which is preceded by the fusion of the apical plasma cell membrane with the LG-limiting membrane [51,161]. Consequently, the penetration of foreign substances via the SC paracellular route, filled with the lamellated lipids attached to CLEs, is unaltered in LKO mice [55,59,137]. In aggregate, as the connotation of its name (“lorica” meaning armor in Latin [62]), or the phylogenetic evidence [12,21] suggest, LOR constitutes an essential element of epidermal host defense, in coordination with the KEAP1/NRF2 signaling pathway [55,58,137]. The adaptor-like function also promotes the structural maturation of corneodesmosomes, SC’s specialized adhesion structure, as extensively studied and suggested previously [141,162,163,164,165].

### 4.4. LOR at the Boundary between Inside and Outside

The reduction and oxidation (redox) state of thiol (–SH)/disulfide (–S–S–) serves as an important indicator of the inside and outside of organisms. The cytosol is highly reduced at the cellular level, owing to a group of thiol-regulating enzymatic systems, including the thioredoxin–thioredoxin reductase and glutaredoxin–glutathione systems [166]. Conversely, the extracellular space is strongly oxidized due to the paucity of the reducing enzymes and the presence of oxygen or other gaseous mediators (such as nitric oxide) [166]. The same may be applied to the epidermal differentiation in which the redox state governs adaptive tissue responses [55,58,136,163] and homeostasis [55,56,57,118,137]. Located in the interstitial space (the paracellular route) of the differentiating epidermal layer are epidermal Langerhans cells (LCs), unique resident leukocytes with a macrophage/dendritic cell (DC) lineage (reviewed in [167,168]). LCs are prototypes of tolerogenic immature DCs [169] that express TJ proteins [170], protect against ACD [171,172], or phorbol ester-induced irritant dermatitis [173], and promote the recovery of the epidermal barrier following physical disruption [174]. The seemingly odd property for classic immune cells may be attributable to epidermal LCs’ peculiar lifestyle, which significantly differs from that of mucosal LCs [175].

### 4.5. Does Epidermal Differentiation Coincide with Immunological Maturation?

**Figure 1:**
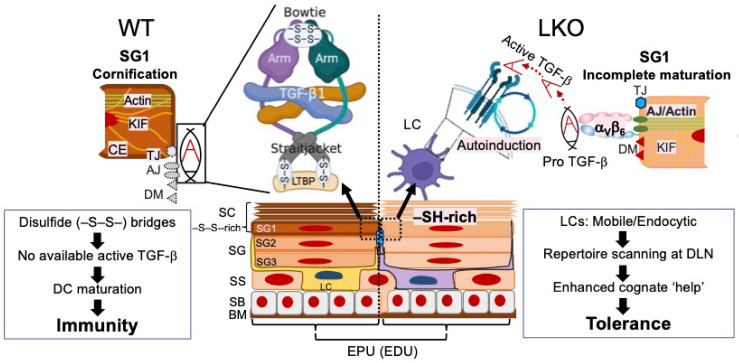
Loricrin (LOR) may directly affect Langerhans cell (LC) differentiation (maturation) and imprint cutaneous immune effector functions. This regulation requires direct interaction between LCs and differentiating keratinocytes (KCs) in the stratum granulosum (SG). LOR anchors differentiate keratins K1/K10 and corneodesmosomes, establishing the desmosome (DM)-keratin (KIF) scaffold in the stratum corneum (SC). LCs are anchored to KCs through integrin α_V_β_6_ expressed on the KC plasma membrane, and activate endogenous transforming growth factor-beta (TGF-β) signaling for epidermal retention. The prodomain (proTGF-β) “fastens” free TGF-β via disulfide (–S–S–) cross-linkages in the bowtie and straitjacket regions. The arm domain anchors keratinocytes via α_V_β_6_ integrin and negatively regulates TGF-β in the extracellular matrix. Therefore, successful cornification may involve disulfide (–S–S–) cross-linkages of the junctional component, possibly inactivating TGF-β permanently and promoting LC maturation. Because LOR promotes the structural maturation of corneodesmosomes (compactization of corneocytes), a delay in the maturation of junctional components would cause the retention of adheren junctions (AJs)/tight junctions (TJs) to the more apical end. The delay in the structural maturation of the LOR-knockout (LKO) epidermis may be advantageous for LCs to remain active (mobile and endocytotic) in situ. The immature structure of the LKO epidermis may imprint adaptive immune responses through more increased TGF-β bioavailability than wild-type (WT) epidermis, causing immunological tolerance (or immune privilege). CE—cornified cell envelope; DC—dendritic cell; BM—basal membrane; SS—stratum spinosum; EPU—epidermal differentiation unit; EDU—epidermal differentiation unit.

Earlier structural observations suggest that epidermal LCs and KCs exist at a constant ratio [176,177], constituting the epidermal proliferation unit (EPU) [176] or the epidermal differentiation unit (EDU) [34]. This strikingly constant ratio between KCs and resient leukocytes (LCs and dendritic epidermal T cells (DETCs)) [178], presumably facilitates the quality control of the differentiating KCs migrating out from the SB [179]. The epidermal residence of LC and DETC requires the transmembrane receptors α_V_β_6_ integrin [180] and α_E_β_7_ integrin (CD103) [181,182], respectively. Therefore, the epidermis serves as a scaffold for the transmembrane communication of these resident leukocytes, and epidermal differentiation (cornification) may efficiently terminate the physical connection. Here, we take LC as a prototype of the epidermal resident leukocyte (Figure 1).

LCs are derived from fetal hematopoietic cells and undergo homeostatic proliferation independent of the blood circulation; however, disrupted LC–KC connection by UV [180,183] evokes the repopulation of blood-borne monocyte-derived LCs to the empty niche [184,185]. Immature LCs stay in the epidermis by activating endogenous TGF-β signaling via α_V_β_6_ integrin expressed on the plasma cell membrane of KCs [180]. Bioactive TGF-β upregulates the adhesion molecules required for epidermal retention, such as the epithelial cell adhesion molecule (EpiCAM) [186,187] and E-cadherin (ECAD) [188]. Conversely, the disrupted integrin-mediated anchorage, not prototypic inflammatory stimuli, such as TIR/nuclear factor kappa B signaling [189,190,191,192], causes LC maturation/migration, causing the transfer of antigens to the T cell-rich inner cortex of local draining lymph nodes (DLNs) [193]. This evidence, along with the fact that the monocyte–macrophage system is sensitive to extracellular redox milieu [194,195], suggests that the epidermal niche is the critical determinant of the epidermal LC phenotype [196,197]. Specifically, the LC connection with KCs [198] through the plasma cell membrane would be mechanically disrupted upon cornification, in which CEs replace the plasma cell membrane of KCs. This deduction leads to our ongoing hypothesis that LOR could profoundly affect LC phenotypes, determined by the expression levels of cell surface proteins [191,199,200], or behavioral responses following physical injury [201]. Indeed, our preliminary observations suggest that LCs in the LKO epidermis exhibit more immature but less migratory (more resident) phenotypes, resulting in the attenuation of the efferent ACD arm, a classic experimental method to determine adaptive immunity (data not shown).

## 5. Conclusions

Summarily, LOR, a strong effector of epidermal differentiation, directly affects LC differentiation (maturation) and thus structurally imprints cutaneous immune effector functions through TGF-β bioavailability regulation. Our hypothesis is rather simple and based on the following evidence and assumptions: 1. epidermal LCs express ECAD/TJ proteins that constitute the “apical junctional complex” and contribute to the formation of the permeability barrier with dynamics, previously proposed as the “reticuloepithelial trap” [202]. 2. The LOR replaces the plasma cell membrane following LG-limiting membrane fusion and promotes corneocyte compactization (maturation) [145]. 3. Delayed structural maturation in the LKO epidermis may retain the α_V_β_6_-mediated LC–KC connection to the more apical end (outside layers). 4. LCs in the LKO epidermis remain mobile and better at capturing antigenic stimuli through endocytosis than LCs in the WT epidermis. 5. The immature structure of the LKO epidermis imprints adaptive immune responses through the relatively increased bioavailability of TGF-β, which may then precondition naïve CD8^+^ T cells [203] and suppress effector T cells during active inflammation [199,200] (Figure 1).

Further investigational studies are required. Revealing this mechanism may provide us with novel therapeutic interventions that percutaneously control immune-mediated diseases, such as multiple sclerosis [204,205] or food allergies [206,207], as an alternative to vaccination against plagues.

## Figures and Tables

**Table 1:** ARCI.

ARCI #	Gene	Function	Category	Reference #
**1**	*TGM1*	Catalyzing ε-(γ-glutamyl) lysine isopeptide bonds	iii	[95]
**2**	*ALOX12B*	Linoleic acid metabolism	i	[93]
**3**	*ALOXE3*	Linoleic acid metabolism	i	[93]
**4**	*ABCA12*	Lipid transport and ceramide linoleic ester formation	ii	[94]
**5**	*CYP4F22*	Catalyzing ultra-long-chain fatty acids	i	[88]
**6**	*NIPAL4*	Mg^2+^ transport	i	[92]
**7**	*N/D*	N/A	N/A	N/A
**8**	*LIPN*	Lipase	i	[91]
**9**	*CERS3*	C24-ceramides synthesis	i	[89]
**10**	*PNPLA1*	Linoleic acid estelification	i	[90]
**11**	*ST14*	Filaggrin processing	iv	[97]
**12**	*CASP14*	Filaggrin processing	iv	[96]
**13**	*SDRC9C7*	Ceramide linoleic ester formation	i	[86]
**14**	*SULT2B1*	Sulfoconjugation of neutral steroids and sterols	i	[87]

Abbreviations: TGM1—transglutaminase 1; ALOX12B—arachidonate 12-lipoxygenase; ALOXE3—arachidonate lipoxygenase 3; ABCA12—ATP-binding cassette subfamily A member 12; CYP4F22—cytochrome P450, family 4, subfamily F, polypeptide 22; NIPAL4—NIPA-like domain-containing 4; LIPN—lipase family member N; CERS3—ceramide synthase 3; PNPLA1—patatin-like phospholipase domain-containing 1; ST14—suppressor of tumorigenicity 14 protein; CASP14—caspase 14; SDRC9C7—short-chain dehydrogenase/reductase family 9C member 7; SULT2B1—sulfotransferase family 2B member 1. N/A: not applicable

**Table 2:** Desmosomal Defects Accompanying Allergic Manifestations.

Clinical Nomenclatures	Gene	Function	Reference #
Netherton syndrome ichthyosis linearis circumflexia	*SPINK5*	Inhibiting serin proteases that degrade corneodesmosomes	[79]
Peeling skin syndrome-1/ Peeling skin disease	*CDSN*	Maintaining corneodesmosomal adhesion	[98]
Skin dermatitis, multiple severe allergies, and metabolic wasting (SAM) syndrome	*DSG1*	Maintaining the desmosomal-keratin scaffold in the differentiating layers	[80]

Abbreviations: SPINK5—serine protease inhibitor of Kasal-type 5; CDSN—corneodesmosin; DSG1—desmoglein 1.

**Table 3:** Ichthyosiform Dermatosis Caused by LOR Mislocalization.

Clinical Nomenclature	Gene	Function	Reference #
Vohwinkel syndrome with ichthyosis	*LOR*	Localizing to the cell periphery and replacing the plasma cell membrane NLS pertubing cornification	[84,103]

Abbreviations: LOR—loricrin; NLS—nuclear localization signal.

## Abbreviations

ACD	allergic contact dermatitis
AD	atopic dermatitis
ARCI	autosomal recessive congenital ichthyosis
Arg	arginine
Ca2+	calcium
CARD14	caspase recruitment domain family member 14
CDSN	corneodesmosin
CE	cornified cell envelope
CIE	congenital ichthyosiform erythroderma
CLE	corneocyte lipid envelopes
DAMP	damage-associated molecular pattern
DC	dendritic cell
DETC	dendritic epidermal T cell
DKO	double knockout
DKS	desmosome-keratin scaffold
DLN	draining lymph node
DMBA	7,12-dimethylbenz(a)anthracene
DNrf2	dominant-negative Nrf2
DSG1	desmoglein 1
ECAD	E-cadherin
EDC	epidermal differentiation complex
EDU	epidermal differentiation unit
EpiCAM	epithelial cell adhesion molecule
EPU	epidermal proliferation unit
FLG	filaggrin
FTT	failure to thrive
HI	harlequin ichthyoses
IFE	interfollicular epidermis
IL	interleukin
ILC	ichthyosis linearis circumflexa
KC	keratinocyte
KEAP1	Kelch-like erythroid cell-derived protein with the cap ‘n’ collar homology-associated protein 1
KG	keratohyalin granules
KLK	kallikrein
LC	Langerhans cell
LCE	late cornified cell envelope proteins
LEKTI	Lympho-epithelial Kazal-type related inhibitor type 5
LG	lamellar granule
LI	lamellar ichthyoses
LKO	loricrin-knockout
LOF	loss-of-function
LOR	loricrin
NMF	natural moisturizing factors
NRF2	nuclear factor erythroid 2-related factor 2
NS	Netherton syndrome
OMIM	Online Mendelian Inheritance in Man
PKK	palmoplantar keratoderma
PSS	peeling skin syndrome
redox	reduction and oxidation
SAM	skin dermatitis, multiple severe allergies, and metabolic wasting
SB	stratum basale
SC	stratum corneum
SCCE	stratum corneum chymotryptic enzyme
SCTE	stratum corneum tryptic enzyme
SG	stratum granulosum
SPINK5	serine protease inhibitor of Kasal-type 5
SPRR	small proline-rich proteins
TEWL	transepidermal water loss
TGF-β	transforming growth factor-beta
TGM	transglutaminase
TH	T helper
TIR	toll-interleukin receptor
TJ	tight junction
UV	ultraviolet
VA	vitamin A
VS	Vohwinkel syndrome
WT	wild type
